# Hypouricemic Effects of *Chrysanthemum indicum* L. and *Cornus officinalis* on Hyperuricemia-Induced HepG2 Cells, Renal Cells, and Mice

**DOI:** 10.3390/plants10081668

**Published:** 2021-08-13

**Authors:** Ok-Kyung Kim, Jeong-Moon Yun, Minhee Lee, Dakyung Kim, Jeongmin Lee

**Affiliations:** 1 Division of Food and Nutrition and Human Ecology Research Institute, Chonnam National University, Gwangju 61186, Korea; 20woskxm@jnu.ac.kr; 2 Department of Medical Nutrition, Kyung Hee University, Yongin 17104, Korea; moon1894@hanmail.net (J.-M.Y.); miniclsrn@khu.ac.kr (M.L.); k4kyung@naver.com (D.K.); 3 Research Institute of Clinical Nutrition, Kyung Hee University, Seoul 02447, Korea

**Keywords:** *Chrysanthemum indicum* L., *Cornus officinalis* Siebold and Zucc., hyperuricemia, uric acid

## Abstract

Hyperuricemia, abnormally excess accumulation of uric acid, is caused by an imbalance between the production and excretion of uric acid and is a major cause of gout. We compared the effects of extracts from *Chrysanthemum indicum* L. (Ci) and *Cornus officinalis* Siebold and Zucc. (Co) on hyperuricemia, both individually and in combination (FSU-CC), using hypoxanthine-treated human liver cancer (HepG2) cells, primary mouse renal proximal tubule cells, and potassium oxonate induced hyperuricemic mice. The Ci contained 7.62 mg/g luteolin and 0 mg/g loganin, Co contained 0 mg/g luteolin and 4.90 mg/g loganin, and FSH-CC contained 3.95 mg/g luteolin and 2.48 mg/g loganin. We found that treatment with Ci, Co, and FSU-CC suppressed the activity of xanthine oxidase and mRNA expression of xanthine dehydrogenase while inducing an increase in the expression levels of the organic anion transporter 1 (OAT1) and organic anion transporter 3 (OAT3) proteins and a decrease in the expression levels of glucose transporter 9 (GLUT9) and urate transporter 1 (URAT1) proteins. Particularly, treatment and supplementation with FSU-CC showed stronger effects than those of supplementation with either Ci or Co alone. We observed that the excretion of creatinine and uric acid in the combination of Ci and Co was higher than that observed in their individual supplementations and was similar to that of the normal group. Therefore, our data suggest that a combination of Ci and Co may potentially be used for the development of effective natural anti-hyperuricemic functional foods.

## 1. Introduction

Purines play important roles as the precursors of nucleic acids, DNA and RNA, promoting the growth, proliferation, and survival of all cells. Purine metabolism is regulated and maintained by the synthesis and degradation of purines, with uric acid being the final compound in purine catabolism [1]. Abnormally excess accumulation of uric acid, known as hyperuricemia, is caused by an imbalance between the production and excretion of uric acid [2]. Consumption of purine-rich and protein-rich foods and alcohol is directly associated with hyperuricemia, which is a major cause of gout due to the deposition of urate crystals in the soft tissues and joints [3]. Gout affects <1 to 6.8% of the population, and the prevalence of hyperuricemia and gout has been increasing over the years [4].

Uric acid is produced by the activities of xanthine oxidase and xanthine dehydrogenase, together referred to as “xanthine oxidoreductase”, in the liver. Xanthine oxidoreductase catalyzes the oxidation of hypoxanthine to xanthine and later to uric acid [5,6]. Xanthine dehydrogenase is initially synthesized and can be converted to xanthine oxidase by reversible sulfhydryl oxidation or irreversible proteolysis [7]. During the production of uric acid, xanthine oxidase delivers electrons directly to molecular oxygen, thus generating the reactive oxygen species (ROS), including the superoxide anion (O_2_^•−^) and hydrogen peroxide (H_2_O_2_), which can further induce oxidative stress [8]. Approximately 75% of uric acid excretion occurs in the kidneys and approximately 25% occurs in the intestines. The circulating uric acid is filtered in the kidneys, and about 90% of the filtered load is usually reabsorbed in the nephrons [9]. Uric acid reabsorption occurs in the renal proximal tubules, facilitated by transporters such as glucose transporter 9 (GLUT9), urate anion transporter 1 (URAT1), and organic anion transporter 4 (OAT4), while uric acid excretion is facilitated by transporters such as organic anion transporter 1 (OAT1) and organic anion transporter 3 (OAT3). Maintaining the function of these uric acid transporters is important to prevent hyperuricemia [10,11].

In this study, we investigated the effects of *Chrysanthemum indicum* L. and *Cornus officinalis* Siebold and Zucc. on hyperuricemia in in vitro and in vivo models. *C. indicum* L., called Indian chrysanthemum, a member of the *Compositae* family, and *C. officinalis* Siebold and Zucc., a member of the *Cornaceae* family, have been reported to exhibit anti-inflammatory and anticancer effects and possess antioxidant properties [12,13,14]. We compared the effects of treatment with extracts from *C. indicum* L. and *C. officinalis* Siebold and Zucc., both individually and in combination, on hypoxanthine-treated human liver cancer cells, primary mouse renal proximal tubule cells, and potassium oxonate induced hyperuricemic mice to develop agents for the prevention of hyperuricemia.

## 2. Materials and Methods

### 2.1. Extract Preparation and HPLC

Flowers of *C. indicum* L. were extracted using water for 8 h at 90 °C. The extract was filtered with Whatman paper No. 6 and concentrated in a rotary evaporator under reduced pressure. The concentrate was lyophilized (Ci) and stored at −20 °C until further use. Fruits of *C. officinalis* Siebold and Zucc. were extracted using water for 8 h at 90 °C. The extract was filtered with Whatman paper No. 6 and concentrated in a rotary evaporator under reduced pressure. The extract was dried using hot air with dextrin (50%) (Co) and stored at −20 °C until further use. Ci and Co were mixed in a ratio of 1:2 (FSH-CC) and stored at −20 °C until further use. Then, these extracts, Ci, Co, and FSH-CC, were analyzed for luteolin and loganin, separately, by high-performance liquid chromatography (HPLC) using an Agilent 1260 Infinity II HPLC system (Santa Clara, CA, USA).

### 2.2. Cell Culture and Treatments

The human liver cancer (HepG2) cells were obtained from the American Type Culture Collection (ATCC; Manassas, VA, USA). The cells were cultured in Dulbecco’s minimal essential medium (DMEM; Hyclone Laboratories, Logan, UT, USA) supplemented with 10% fetal bovine serum (FBS; Hyclone Laboratories), 100 mg/L penicillin–streptomycin, and 2 mmol/L glutamine (Hyclone Laboratories) at 37 °C in a humid atmosphere of 5% carbon dioxide (CO_2_).

To obtain the primary mouse renal proximal tubule cells, kidney was isolated from Balb/c mice (22–25 g, 6 weeks, male). The kidney was minced using Hank’s balanced salt solution (HBSS) containing trypsin, with the addition of 1 mg/mL deoxyribonuclease (DNAse) and 2 mg/mL collagenase type I (Sigma-Aldrich, St. Louis, MO, USA). After 30 min, the solution was passed through an 80-mesh and 1709-mesh sieve (Fisher Scientific, Pittsburgh, PA, USA) to remove the cell debris and glomeruli. Proximal tubule cells remained on the sieve filter and were collected by washing the sieve filter with HBSS. The proximal tubule cell suspension was centrifuged for 10 min at 1000 revolutions per minute (rpm) at 4 °C, and the cell pellet was collected.

HepG2 cells and primary mouse renal proximal tubule cells were cultured with Ci, Co, and FSH-CC for 24 h and treated with 4 mM hypoxanthine. After 2 h, assays were performed to measure the activity of xanthine oxidase; mRNA expression of xanthine dehydrogenase; and the expression levels of OAT1, OAT3, GLUT9, and URAT1 proteins.

### 2.3. Animals

The Institutional Animal Care and Use Committee of Kyung Hee University approved the protocol (KHGASP-20–410) for the use of animals in this study. The animals were cared for in accordance with the “Guidelines for Animal Experiments” established by the university.

Six-week-old male C57 black 6 (C57BL6) mice were purchased from SaeRon Bio (Uiwang, Korea) and housed in cages under automatically controlled temperature (22 ± 2 °C), humidity (about 50%), and lighting (12:12-h light-dark cycle) conditions. The mice in the control group with normal diet were fed a commercial pelleted chow (AIN-93G rodent purified diet, Orient Bio, Korea) and water ad libitum. All the mice were randomly divided into eight groups of eight mice per group as follows: normal control (NC), control (C; hyperuricemia-induced mice), positive control (PC; hyperuricemia-induced mice with oral supplementation of allopurinol, xanthine oxidase inhibitor, 10 mg/kg body weight (b.w.)), Ci 300 (hyperuricemia-induced mice with oral supplementation of Ci, 300 mg/kg b.w.), Co 300 (hyperuricemia-induced mice with oral supplementation of Co, 300 mg/kg b.w.), FSH-CC 150 (hyperuricemia-induced mice with oral supplementation of FSH-CC, 150 mg/kg b.w.), FSH-CC 300 (hyperuricemia-induced mice with oral supplementation of FSH-CC, 300 mg/kg b.w.), and FSH-CC 600 (hyperuricemia-induced mice with oral supplementation of FSH-CC, 600 mg/kg b.w.). The extracted samples were orally administered for 21 days. To induce hyperuricemia, an intraperitoneal injection of 200 mg/kg b.w. potassium oxonate (Sigma-Aldrich Co, St. Louis, MO, USA) was given. After 2 h, the mice’s urine was collected and the mice were anesthetized with isoflurane.

### 2.4. Levels of Uric Acid and Creatinine in the Urine and Serum

Blood was centrifuged at 3000 rpm for 10 min and the serum was separated. The levels of uric acid in the urine and serum were determined using the uric acid assay kits (BioVision Inc., Milpitas, CA, USA), while the levels of creatinine in the urine and serum were determined using the creatinine assay kit (BioVision Inc., Milpitas, CA, USA).

### 2.5. Activity of Xanthine Oxidase

The activity of xanthine oxidase was determined from the HepG2 cells in the culture medium and serum from mice using the Xanthine Oxidase Activity Assay Kit (Sigma-Aldrich Co, St. Louis, MO, USA).

### 2.6. mRNA Expression of Xanthine Dehydrogenase

mRNA was extracted from the HepG2 cells and liver tissues using the RNeasy Mini Kit (QIAGEN, MD, USA). Synthesis of complementary DNA (cDNA) using mRNA was performed using the iScript cDNA Synthesis Kit (Bio-Rad Laboratories, Inc., Hercules, CA, USA). Polymerase chain reaction (PCR) consisted of 40 cycles of denaturation (95 °C for 15 s), annealing (58 °C for 15 s), and extension (72 °C for 30 s) using the SYBR Green PCR Master Mix (iQ SYBR Green Supermix; Bio-Rad Laboratories, Inc.) and the following primers: GAPDH (H) forward primer 5′-CCC CAC ACA CAT GCA CTT ACC-3′, reverse primer 5′-TTG CCA AGT TGC CTG TCC TT-3′; xanthine dehydrogenase (H) forward primer 5′-ATT GGT GCT GTG GTT GCT-3′, reverse primer 5′-TGT GAT AAT GGC TGG TAG TTC TTC; GAPDH (R) forward primer 5′-CAT GGC CTT CCG TGT TCC TA-3′, reverse primer 5′-GCG GCA CGT CAG ATC CA-3′; xanthine dehydrogenase (R) forward primer 5′-TGC GAA GGA TGA GGT TAC T-3′, reverse primer 5′-GGA TTG TGA TAA TGG CTG GAA-3′. The cDNA was amplified using the real-time PCR detection system (Bio-Rad, Hercules, CA, USA), and data analysis was performed using the CFX Maestro Analysis Software (Bio-Rad Laboratories, Inc.).

### 2.7. Serum Triglycerides, Cholesterols, Aspartate Transaminase (AST), and Alanine Transaminase (ALT)

Concentrations of total triglycerides (TG), total cholesterol (TC), very-low-density lipoprotein (VLDL)/low-density lipoprotein (LDL) cholesterol, high-density lipoprotein (HDL) cholesterol, aspartate transaminase (AST), and alanine transaminase (ALT) in serum were determined by enzyme-linked colorimetric methods using commercial kits (BioVision Inc., Milpitas, CA, USA).

### 2.8. Antioxidant Enzyme Activity in the Liver

The liver tissues were lysed using the CelLytic MT lysis reagent (Sigma), and the antioxidant enzyme activity was measured using the superoxide dismutase (SOD), catalase (CAT), and glutathione peroxidase (GPx) assay kits (Biomax Inc., Seoul). Malondialdehyde (MDA), a lipid peroxidation marker, was measured using MDA assay kits (BioVision Inc., Milpitas, CA, USA).

### 2.9. Expression Levels of OAT1, OAT3, GLUT9, and URAT1 Proteins

The primary mouse renal tubular epithelial cells and kidney tissues were lysed using the CelLytic MT lysis reagent. Equal amounts of proteins (100 μg/lane) were separated by electrophoresis using 10% Mini-PROTEAN TGX Precast Gels (Bio-Rad Laboratories, Inc.) and transferred to polyvinylidene difluoride membranes using Trans-Blot Turbo Transfer System (Bio-Rad Laboratories, Inc.). The membranes were incubated for 1 h in a blocking solution containing 5% nonfat milk in Tris-buffered saline and further incubated for 12 h at 4 °C with the antibodies recognizing OAT1 (1:500; MyBioSource), OAT3 (1:1000; Santa Cruz), GLUT9 (1:800; Invitrogen), URAT1 (1:800; MyBioSource), and β-actin (1:1000; Cell Signaling Technology). Thereafter, the membranes were incubated with secondary antibodies (anti-rabbit immunoglobulin G (IgG) horseradish peroxidase (HRP)-linked antibody, 1:3000; Cell Signaling Technology, Inc.) for 1 h at room temperature. The immunoreactive protein bands were detected using EzWestLumi plus (ATTO, Tokyo, Japan) and analyzed using Ez-Capture II (ATTO) and CS Analyzer v.3.0 (ATTO).

### 2.10. Statistical Analysis

All data are presented as mean ± standard deviation (SD). The data were statistically evaluated using Duncan’s multiple range tests after one-way analysis of variance (ANOVA) using SPSS statistical procedures (SPSS PASW Statistic v.23.0, SPSS Inc., Chicago, IL, USA). When the data were subjected to prior investigations before analysis, parametric assumptions including homoscedasticity and normality of observations were satisfied. Differences were considered to be statistically significant at *p* < 0.05 level.

## 3. Results

### 3.1. Luteolin and Loganin of Ci, Co, and FSH-CC

**Figure 1:**
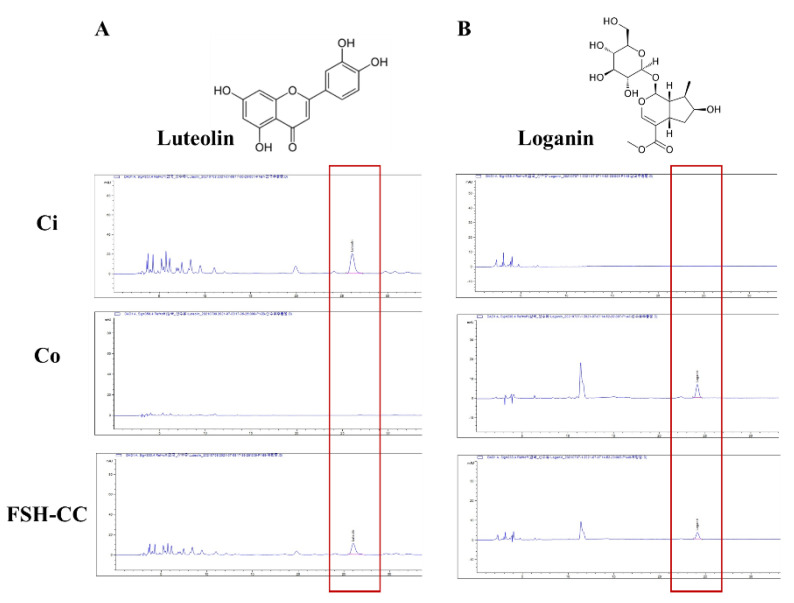
High-performance liquid chromatography analysis of luteolin (**A**) and loganin (**B**) levels in Ci, Co, and FSH-CC.

The HPLC analysis of the Ci, Co, and FSH-CC revealed three peaks matching those of the commercial standards luteolin (Figure 1A) and loganin (Figure 1B). The Ci contained 7.62 mg/g luteolin and 0 mg/g loganin, Co contained 0 mg/g luteolin and 4.90 mg/g loganin, and FSH-CC contained 3.95 mg/g luteolin and 2.48 mg/g loganin.

### 3.2. The Combination of Ci and Co Suppressed the Xanthine Oxidase Activity and Xanthine Dehydrogenase mRNA Expression in Liver Cells More Than Their Individual Treatments

**Figure 2:**
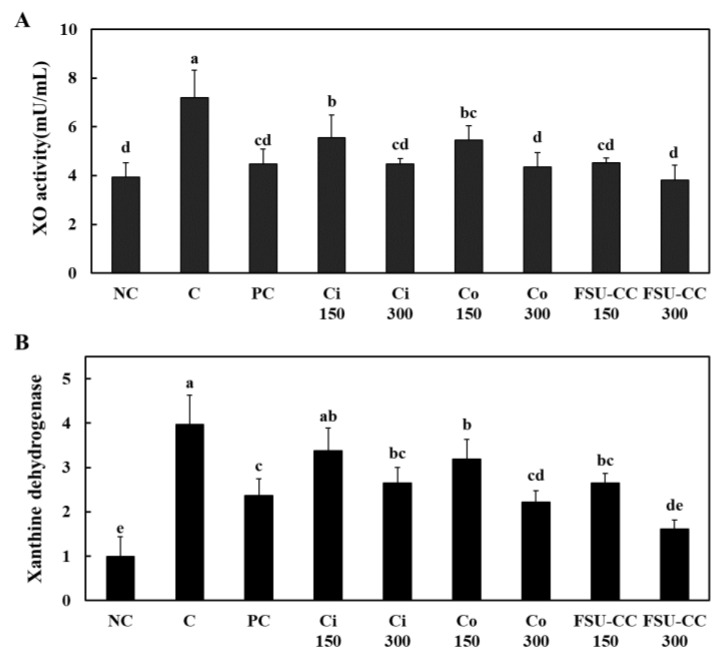
Xanthine oxidase activity (**A**) and xanthine dehydrogenase mRNA expression (**B**) in hypoxanthine-treated human liver cancer (HepG2) cells, with and without Ci, Co, or FSU-CC. NC: normal control, C: 4 mM hypoxanthine, PC: 4 mM hypoxanthine + 100 μM allopurinol, Ci 150: 4 mM hypoxanthine + 150 μg/mL *Chrysanthemum indicum* L., Ci 300: 4 mM hypoxanthine + 300 μg/mL *C. indicum* L., Co 150: 4 mM hypoxanthine + 150 μg/mL *Cornus officinalis* Siebold and Zucc., Co 300: 4 mM hypoxanthine + 300 μg/mL *C. officinalis* Siebold and Zucc., FSU-CC 150: 4 mM hypoxanthine + 150 μg/mL mixture of *C. indicum* L. and *C. officinalis* Siebold and Zucc. (1:2), FSU-CC 300: 4 mM hypoxanthine + 300 μg/mL mixture of *C. indicum* L. and *C. officinalis* Siebold and Zucc. (1:2). Different letters indicate significant difference at *p* < 0.05, as determined by Duncan’s multiple range test.

We found that hypoxanthine treatment (C) increased the activity of xanthine oxidase and mRNA expression of xanthine dehydrogenase as compared with those in the normal control (NC). However, Ci and Co treatments revealed a significant decrease in the activity of xanthine oxidase and mRNA expression of xanthine dehydrogenase as compared with those in the control group. In addition, the combination of Ci and Co (FSU-CC) suppressed xanthine oxidase activity and xanthine dehydrogenase mRNA expression more than the individual treatment with either Ci or Co alone. Moreover, FSU-CC 300 treatment resulted in the most significant reduction in the xanthine oxidase activity and xanthine dehydrogenase mRNA expression among all the hypoxanthine-treated HepG2 cells (*p* < 0.05) (Figure 2).

### 3.3. The Combination of Ci and Co Increased the Expression Levels of OAT1 and OAT3 and Suppressed the Expression Levels of GLUT9 and URAT1 in Renal Proximal Tubule Cells More Than Their Individual Treatments

**Figure 3:**
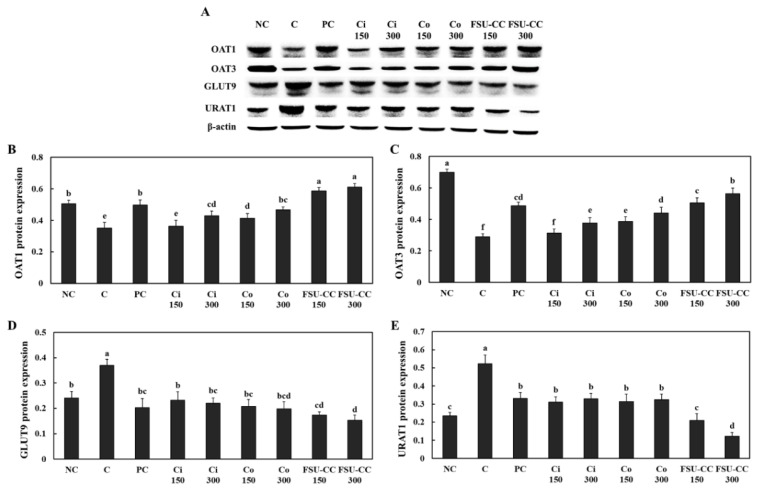
Expression levels of proteins by Western blotting (band images (**A**), relative protein expression (**B**–**E**)) in the hypoxanthine-treated primary mouse renal proximal tubule cells, with and without Ci, Co, or FSU-CC. NC: normal control, C: 4 mM hypoxanthine, PC: 4 mM hypoxanthine + 100 μM allopurinol, Ci 150: 4 mM hypoxanthine + 150 μg/mL *C. indicum* L., Ci 300: 4 mM hypoxanthine + 300 μg/mL *C. indicum* L., Co 150: 4 mM hypoxanthine + 150 μg/mL *C. officinalis* Siebold and Zucc., Co 300: 4 mM hypoxanthine + 300 μg/mL *C. officinalis* Siebold and Zucc., FSU-CC 150: 4 mM hypoxanthine + 150 μg/mL mixture of *C. indicum* L. and *C. officinalis* Siebold and Zucc. (1:2), FSU-CC 300: 4 mM hypoxanthine + 300 μg/mL mixture of *C. indicum* L. and *C. officinalis* Siebold and Zucc. (1:2). Different letters indicate significant difference at *p* < 0.05, as determined by Duncan’s multiple range test.

We confirmed the uric acid excretion transporters, OAT1 and OAT3, as well as the uric acid reabsorption transporters, GLUT9 and URAT1, in the primary mouse renal proximal tubule cells. Hypoxanthine treatment in these cells induced a decrease in the expression levels of OAT1 and OAT3 compared with those in the normal control group, while Ci and Co treatment groups exhibited increased expression levels of OAT1 and OAT3 compared with those in the hypoxanthine control group. Moreover, the combination of Ci and Co (FSU-CC) increased the expression levels of OAT1 and OAT3 more than the individual treatment with either Ci or Co alone (*p* < 0.05) (Figure 3B,C).

Compared to the normal control group, hypoxanthine treatment induced an increase in the expression levels of GLUT9 and URAT1 in the primary mouse renal proximal tubule cells, while Ci and Co treatment groups exhibited significantly decreased expression levels of GLUT9 and URAT1 compared with those in the hypoxanthine treatment control group. Moreover, the combination of Ci and Co (FSU-CC) decreased expression levels of GLUT9 and URAT1 more than the individual treatment with either Ci or Co alone (*p* < 0.05) (Figure 3D,E).

### 3.4. The Combination of Ci and Co Increased the Excretion of Creatinine and Uric Acid in Hyperuricemia-Induced Mice More Than Their Individual Treatments

**Figure 4:**
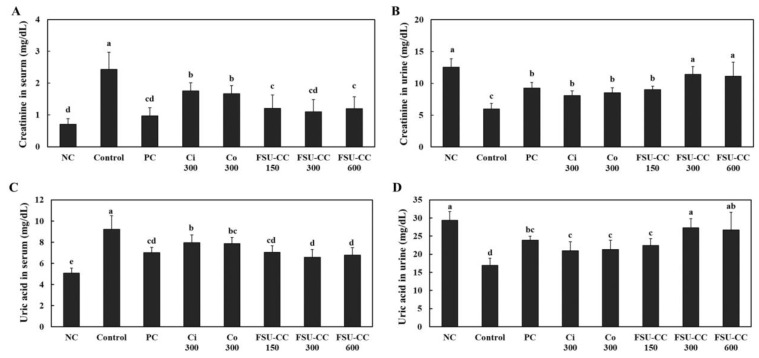
Levels of creatinine in serum (**A**) and urine (**B**) and uric acid in serum (**C**) and urine (**D**) in the hyperuricemia-induced mice supplemented with and without Ci, Co, or FSU-CC. NC: normal control, C: hyperuricemia-induced mice, PC: hyperuricemia-induced mice with oral supplementation of allopurinol 10 mg/kg b.w., Ci 300: hyperuricemia-induced mice with oral supplementation of *C. indicum L.* 300 mg/kg b.w., Co 300: hyperuricemia-induced mice with oral supplementation of *C. officinalis* Siebold and Zucc. 300 mg/kg b.w., FSU-CC 150: hyperuricemia-induced mice with oral supplementation of mixture of *C. indicum* L. and *C. officinalis* Siebold and Zucc. (1:2) 150 mg/kg b.w., FSU-CC 300: hyperuricemia-induced mice with oral supplementation of mixture of *C. indicum* L. and *C. officinalis* Siebold and Zucc. (1:2) 300 mg/kg b.w., FSU-CC 600: hyperuricemia-induced mice with oral supplementation of mixture of *C. indicum* L. and *C. officinalis* Siebold and Zucc. (1:2) 600 mg/kg b.w. Different letters indicate significant difference at *p* < 0.05, as determined by Duncan’s multiple range test.

We investigated the effects of Ci and Co supplementation on hyperuricemia-induced mice and found that Ci and Co supplementation did not affect the change in the levels of serum ALT, AST, triglycerides, total cholesterol, HDL cholesterol, and LDL cholesterol in hyperuricemia-induced mice (Table 1). We measured the levels of creatinine and uric acid in the serum and urine in hyperuricemia-induced mice to confirm whether Ci and Co supplementation affects the excretion of creatinine and uric acid in them. Compared to the normal control, hyperuricemia-induced mice showed a significant increase in the levels of creatinine and uric acid in the serum and a significant decrease in the levels of creatinine and uric acid in the urine. Ci 300 and Co 300 supplementation groups exhibited significantly decreased levels of creatinine and uric acid in the serum and increased levels of creatinine and uric acid in the urine as compared with those in the control group. FSU-CC 300 decreased the levels of creatinine and uric acid in the serum and increased the levels of creatinine and uric acid in the urine more than the individual supplementation of either Ci 300 or Co 300 alone (*p* < 0.05) (Figure 4).

### 3.5. The Combination of Ci and Co Increased the Xanthine Oxidase Activity and Xanthine Dehydrogenase mRNA Expression and Inhibited the Oxidative Stress in Hyperuricemia-Induced Mice More Than Their Individual Treatments

**Figure 5:**
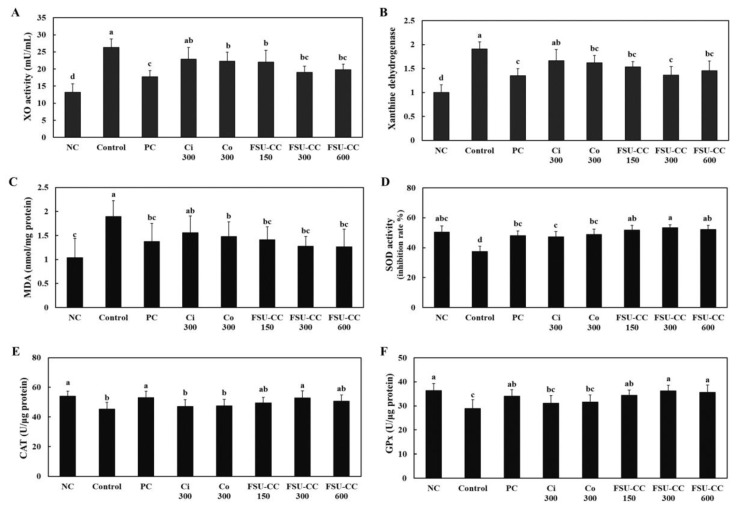
Xanthine oxidase activity (**A**); xanthine dehydrogenase mRNA expression (**B**); malondialdehyde (MDA) levels (**C**); and activities of superoxide dismutase (SOD) (**D**), catalase (CAT) (**E**), and glutathione peroxidase (GPx) (**F**) in the liver from hyperuricemia-induced mice supplemented with and without Ci, Co, or FSU-CC. NC: normal control, C: hyperuricemia-induced mice, PC: hyperuricemia-induced mice with oral supplementation of allopurinol 10 mg/kg b.w., Ci 300: hyperuricemia-induced mice with oral supplementation of *C. indicum L.* 300 mg/kg b.w., Co 300: hyperuricemia-induced mice with oral supplementation of *C. officinalis* Siebold and Zucc. 300 mg/kg b.w., FSU-CC 150: hyperuricemia-induced mice with oral supplementation of mixture of *C. indicum* L. and *C. officinalis* Siebold and Zucc. (1:2) 150 mg/kg b.w., FSU-CC 300: hyperuricemia-induced mice with oral supplementation of mixture of *C. indicum* L. and *C. officinalis* Siebold and Zucc. (1:2) 300 mg/kg b.w., FSU-CC 600: hyperuricemia-induced mice with oral supplementation of mixture of *C. indicum* L. and *C. officinalis* Siebold and Zucc. (1:2) 600 mg/kg b.w. Different letters indicate significant difference at *p* < 0.05, as determined by Duncan’s multiple range test.

We measured the xanthine oxidase activity, xanthine dehydrogenase mRNA expression, and oxidative stress in the liver of hyperuricemia-induced mice. The xanthine oxidase activity and xanthine dehydrogenase mRNA expression in the liver were significantly increased in the hyperuricemia-induced mice group compared with those in the normal control group. However, Ci 300 and Co 300 supplementation suppressed the xanthine oxidase activity and xanthine dehydrogenase mRNA expression in the liver of hyperuricemia-induced mice. FSU-CC 300 supplementation significantly decreased the xanthine oxidase activity and xanthine dehydrogenase mRNA expression in the liver of hyperuricemia-induced mice more than the individual supplementation of either Ci 300 or Co 300 alone (*p* < 0.05) (Figure 5A,B).

We found that hyperuricemia-induced mice exhibited an increase in the MDA levels and a decrease in the activities of antioxidant enzymes, including SOD, CAT, and GPx, compared with those in the normal control group. Ci 300, Co 300, and FSU-CC supplementation induced a decrease in the MDA levels and an increase in the antioxidant enzyme activities compared with those in the control group (*p* < 0.05) (Figure 5C–F).

### 3.6. The Combination of Ci and Co Increased the Expression Levels of OAT1 and OAT3 and Suppressed the Expression Levels of GLUT9 and URAT1 in Hyperuricemia-Induced Mice More Than Their Individual Treatments

**Figure 6:**
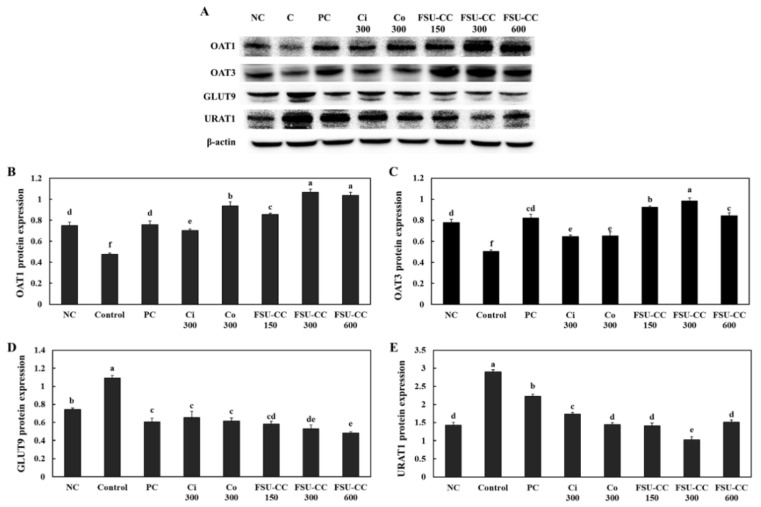
Expression levels of proteins by Western blotting (band images (**A**), relative protein expression (**B**–**E**)) in the kidney of hyperuricemia-induced mice supplemented with and without Ci, Co, or FSU-CC. NC: normal control, C: hyperuricemia-induced mice, PC: hyperuricemia-induced mice with oral supplementation of allopurinol 10 mg/kg b.w., Ci 300: hyperuricemia-induced mice with oral supplementation of *C. indicum L.* 300 mg/kg b.w., Co 300: hyperuricemia-induced mice with oral supplementation of *C. officinalis* Siebold and Zucc. 300 mg/kg b.w., FSU-CC 150: hyperuricemia-induced mice with oral supplementation of mixture of *C. indicum* L. and *C. officinalis* Siebold and Zucc. (1:2) 150 mg/kg b.w., FSU-CC 300: hyperuricemia-induced mice with oral supplementation of mixture of *C. indicum* L. and *C. officinalis* Siebold and Zucc. (1:2) 300 mg/kg b.w., FSU-CC 600: hyperuricemia-induced mice with oral supplementation of mixture of *C. indicum* L. and *C. officinalis* Siebold and Zucc. (1:2) 600 mg/kg b.w. Different letters indicate significant difference at *p* < 0.05, as determined by Duncan’s multiple range test.

Hyperuricemia-induced mice showed a decrease in the expression levels of OAT1 and OAT3 in the kidney compared with those in the normal control group. Ci 300 and Co 300 supplementation increased the expression levels of OAT1 and OAT3 in the kidney of hyperuricemia-induced mice compared with those in the control group. In addition, FSU-CC 300 supplementation significantly increased the expression levels of OAT1 and OAT3 in the kidney more than the individual supplementation of either Ci 300 or Co 300 alone (*p* < 0.05) (Figure 6B,C).

Hyperuricemia-induced mice exhibited an increase in the expression levels of GLUT9 and URAT1 in the kidney as compared to the normal control. Ci 300 and Co 300 supplementation significantly decreased the expression levels of GLUT9 and URAT1 in the kidney compared with those in the control group. Moreover, FSU-CC 300 decreased the expression levels of GLUT9 and URAT1 in the kidney of hyperuricemia-induced mice more than the individual supplementation of either Ci 300 or Co 300 alone (*p* < 0.05) (Figure 6D,E).

## 4. Discussion

Recently, the prevalence of hyperuricemia-induced gout has been increasing, and the treatment for gout includes the use of nonsteroidal anti-inflammatory drugs to relieve the symptoms of the illness and the use of allopurinol and xanthine oxidase inhibitors to reduce the production of uric acid [15]. However, these drugs have side effects, including gastrointestinal toxicity and bleeding, renal toxicity, and hypersensitivity reactions [16]. Therefore, alternative therapies have been explored in an attempt to treat and prevent hyperuricemia [17,18]. We compared the effects of Ci, Co, and a combination of Ci and Co (FSU-CC) on hyperuricemia-induced HepG2 cells, primary mouse renal proximal tubule cells, and potassium oxonate induced hyperuricemic mice. The flowers of *C. indicum* L. and fruits of *C. officinalis* Siebold and Zucc. are widely known for their health benefits as traditional tea and are accepted for use as food in Korea [19,20]. The present study aimed to compare the effects of Ci, Co, and a combination and to develop agents for the prevention of hyperuricemia.

Xanthine oxidase, converted from xanthine dehydrogenase, acts as a key enzyme for the oxidation of hypoxanthine and xanthine during the production of uric acid in the liver. It is well known that the mitochondrial ROS are produced during the xanthine oxidase mediated production of uric acid [7,8]. We found an increase in the xanthine oxidase activity and xanthine dehydrogenase mRNA expression in hypoxanthine-treated HepG2 cells and the liver of potassium oxonate induced hyperuricemic mice as compared to that in the normal cells and liver of healthy mice. In addition, the potassium oxonate induced hyperuricemic mice exhibited oxidative stress in the liver due to a decrease in antioxidant enzyme activities and increase in MDA levels. However, treatment with Ci, Co, and FSU-CC suppressed the xanthine oxidase activity, xanthine dehydrogenase mRNA expression, and oxidative stress in hypoxanthine-treated HepG2 cells and the liver of potassium oxonate induced hyperuricemic mice. We found that the combination of Ci and Co inhibited the activity of xanthine oxidase more than either of the two given separately.

The study of Nishida [21] has demonstrated the significant positive correlations between the excretion of creatinine and uric acid in urine. Thus, we measured the levels of creatinine and uric acid in the serum and urine to observe the excretion process of creatinine and uric acid. Supplementation of Ci, Co, and FSU-CC increased the excretion of creatinine and uric acid through urine, which was suppressed by potassium oxonate injection in mice. Moreover, the excretion of creatinine and uric acid in the combination of Ci and Co was higher than that of individual supplementation and was similar to that of the normal group. These results indicate that the combination of Ci and Co helps to treat hyperuricemia more than individual dietary supplements.

In order to elucidate the mechanisms of Ci, Co, and FSU-CC that mediate the excretion of uric acid in hyperuricemia, we observed the expression of the transporters involved in uric acid excretion in hypoxanthine-treated primary mouse renal proximal tubule cells and the kidney from potassium oxonate induced hyperuricemic mice. Previous studies have identified GLUT9 and URAT1, involved in uric acid reabsorption, and OAT1 and OAT3, involved in uric acid excretion, as potential therapeutic targets for hyperuricemia [10,22,23]. We have shown in the present study that Ci, Co, and FSU-CC significantly increased the expression levels of OAT1 and OAT3 while decreasing the expression levels of GLUT9 and URAT1 in hypoxanthine-treated primary mouse renal proximal tubule cells and the kidney from potassium oxonate induced hyperuricemic mice. Moreover, the combination of Ci and Co increased the expression levels of OAT1 and OAT3 and suppressed the expression levels of GLUT9 and URAT1 more than the individual treatments. Therefore, we hypothesize that FSU-CC aids in maintaining the function of these uric acid transporters to prevent hyperuricemia more than the individual treatment with either Ci or Co alone.

We showed that Ci contained luteolin, a common flavonoid, and Co contained loganin, an iridoid glycoside. Matsuda et al. isolated new flavanone glycosides and a phenylbutanoid glycoside from the flowers of *C. indicum* L. and found inhibitory activity for rat lens aldose reductase [24]. Dong et al. showed the various pharmacological activities of the *C. officinalis* Siebold and Zucc. extract and found chemical compounds, including terpenoids, flavonoids, and tannin, identified from *C. officinalis* Siebold and Zucc. [20]. Several studies have demonstrated that treatment with phenolic compounds suppresses the development of hyperuricemia in vitro, in vivo, and in human clinical trial studies [25,26,27]. According to these reports and our present results, we can assume that phenolic compounds from Ci and Co can have hypouricemic effects on hyperuricemia-induced HepG2 cells, renal cells, and mice. However, further human clinical trials are needed to fully understand the effects of dietary supplementation with both Ci and Co on hyperuricemia.

## 5. Conclusions

**Figure 7:**
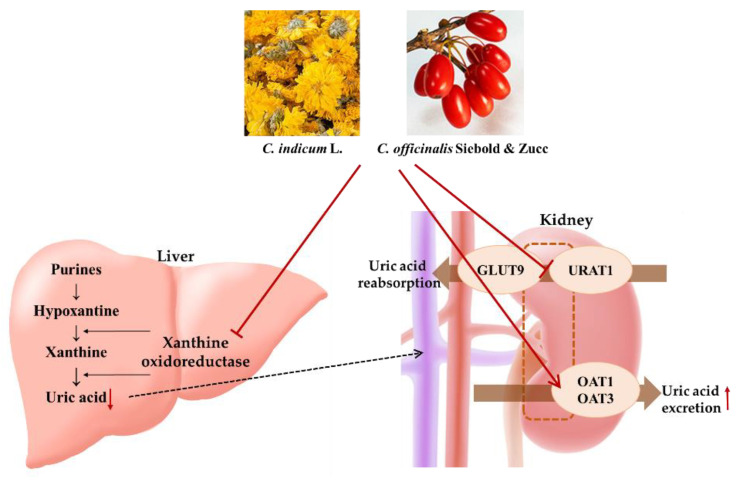
Schematic representation of the effect of Ci, Co, or FSU-CC on hyperuricemia. Ci, Co, or FSU-CC inhibited the production of uric acid in the liver and excretion of uric acid in the renal cells.

We compared the effects of extracts from Ci, Co, and FSU-CC on hyperuricemia using hypoxanthine-treated HepG2 cells, primary mouse renal proximal tubule cells, and potassium oxonate induced hyperuricemic mice. We found that the FSU-CC treatment inhibited the production and excretion of uric acid more than the individual treatment with either Ci or Co alone in both in vitro and in vivo models. We confirmed that treatment with FSU-CC directly inhibited the production of uric acid in hepatocytes and increased the expression of uric acid transporters in renal cells, which are involved in the excretion process (Figure 7). This study provides scientific evidence and describes the underlying mechanisms responsible for the anti-hyperuricemic effects of Ci and Co. Therefore, our data suggest that a combination of Ci and Co may potentially be used for the development of effective natural anti-hyperuricemic agents.

## Figures and Tables

**Table 1:** Changes in the levels of serum alanine transaminase (ALT), aspartate transaminase (AST), triglycerides, total cholesterol, high-density lipoprotein (HDL) cholesterol, and low-density lipoprotein (LDL) cholesterol in the hyperuricemia-induced mice supplemented with and without Ci, Co, or FSU-CC.

	AST (mU/mL)	ALT (mU/mL)	Triglycerides (μg/mL)	Total cholesterol (μg/mL)	HDL (μg/mL)	LDL (μg/mL)
NC	22.79 ± 3.34^ns^	3.37 ± 0.41^ns^	2.60 ± 0.39^ns^	7.58 ± 0.72^ns^	1.72 ± 0.24^ns^	1.63 ± 0.89^ns^
C	23.02 ± 2.26	2.91 ± 0.64	2.26 ± 0.14	8.06 ± 0.81	1.89 ± 0.29	1.74 ± 0.84
PC	21.83 ± 2.49	2.91 ± 0.35	2.62 ± 0.26	7.75 ± 0.83	1.70 ± 0.34	1.30 ± 0.91
Ci 300	23.66 ± 2.13	3.06 ± 0.77	2.63 ± 0.37	7.56 ± 0.97	1.74 ± 0.32	1.61 ± 0.75
Co 300	23.39 ± 2.18	3.13 ± 0.48	2.44 ± 0.33	8.33 ± 0.56	1.75 ± 0.19	1.55 ± 092
FSU-CC 150	22.77 ± 1.29	2.79 ± 0.32	2.47 ± 0.42	7.60 ± 0.76	1.80 ± 0.33	1.30 ± 1.55
FSU-CC 300	24.08 ± 6.82	3.25 ± 0.58	2.43 ± 0.14	7.72 ± 1.46	1.57 ± 0.22	1.38 ± 1.32
FSU-CC 600	22.85 ± 1.36	3.24 ± 0.78	2.61 ± 0.19	7.11 ± 0.56	1.64 ± 0.27	1.24 ± 0.79

^ns^ not significant. NC: normal control, C: hyperuricemia-induced mice, PC: hyperuricemia-induced mice with oral supplementation of allopurinol 10 mg/kg b.w., Ci 300: hyperuricemia-induced mice with oral supplementation of *C. indicum L.* 300 mg/kg b.w., Co 300: hyperuricemia-induced mice with oral supplementation of *C. officinalis* Siebold and Zucc. 300 mg/kg b.w., FSU-CC 150: hyperuricemia-induced mice with oral supplementation of mixture of *C. indicum* L. and *C. officinalis* Siebold and Zucc. (1:2) 150 mg/kg b.w., FSU-CC 300: hyperuricemia-induced mice with oral supplementation of mixture of *C. indicum* L. and *C. officinalis* Siebold and Zucc. (1:2) 300 mg/kg b.w., FSU-CC 600: hyperuricemia-induced mice with oral supplementation of mixture of *C. indicum* L. and *C. officinalis* Siebold and Zucc. (1:2) 600 mg/kg b.w. Different letters indicate significant difference at *p* < 0.05, as determined by Duncan’s multiple range test.

## Data Availability

Not applicable.
